# Engineering a conditionally active cetuximab prodrug via affibody-based paratope masking

**DOI:** 10.1186/s13036-026-00705-1

**Published:** 2026-06-08

**Authors:** Anna Mestre Borras, Hanna Mehari, Athanasios Bitzios, Ivan Zelepukin, Moeen Ud-Din, Tianqi Xu, Sihan Li, Anna Orlova, Vladimir Tolmachev, Stefan Ståhl, Anzhelika Vorobyeva, John Löfblom

**Affiliations:** 1 Department of Protein Science, School of Engineering Sciences in Chemistry, Biotechnology and Health, KTH Royal Institute of Technology, Stockholm, Sweden; 2 Department of Immunology, Genetics and Pathology, Uppsala University, Uppsala, 751 85 Sweden; 3 Department of Medicinal Chemistry, Uppsala University, Uppsala, 751 83 Sweden; 4 Science for Life Laboratory, Uppsala, 751 83 Sweden

**Keywords:** Cetuximab, Prodrug, Affibody molecule, Conditional activation, Matriptase, Antibody engineering

## Abstract

The application of monoclonal antibodies as targeted drugs has revolutionized cancer therapy, yet their efficacy is sometimes limited by toxicities arising from target expression in healthy tissues. Various strategies have emerged to overcome these issues, including the design of antibody-based prodrugs. Here, we report the generation of affibody-based masking domains designed to specifically bind and mask the paratope of the anti-epidermal growth factor receptor (EGFR) antibody, cetuximab. Bacterial display of affibody library and cell sorting were employed to isolate suitable candidates. Both experimental and computational analyses confirmed that several of these selected affibodies effectively blocked cetuximab from binding EGFR. A cetuximab prodrug was created by fusing the most promising masking candidate to the antibody heavy chains. The prodrug demonstrated over 400-fold difference in growth inhibition effect between its masked and unmasked states in vitro. A biodistribution study comparing cetuximab and its prodrug demonstrated the feasibility of conditional activation of the cetuximab prodrug in a xenograft mouse model. This study is the first to describe the use of an affibody masking domain to create an antibody-based prodrug, demonstrating significant potential for further development of safer and more efficacious cancer therapies.

**Supplementary Information:**

The online version contains supplementary material available at 10.1186/s13036-026-00705-1.

## Introduction

**Fig. 1:**
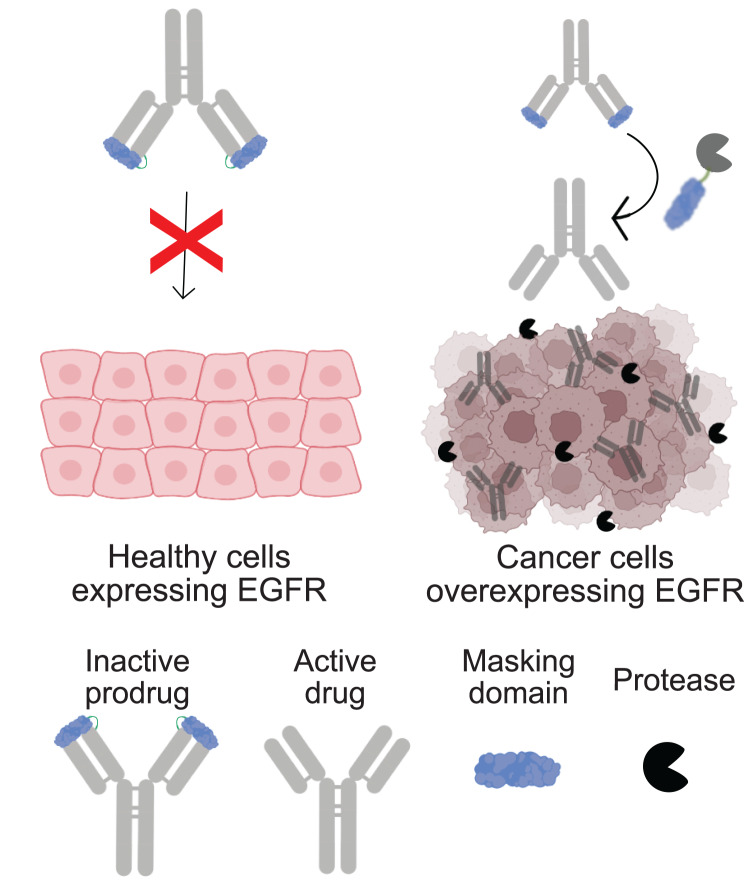
Protease-activated antibody prodrug mechanism. In healthy tissues that express EGFR, the antibody prodrug remains inactive due to steric blocking by affibody-based masking domains, preventing receptor engagement. In contrast, in the tumor microenvironment, elevated levels of tumor-associated proteases cleave the masking domains, converting the prodrug into its active form thus allowing EGFR binding on cancer cells

Monoclonal antibodies have revolutionized cancer therapy by offering targeting of tumor-associated antigens, with reduced systemic toxicity compared to traditional chemotherapeutics [1]. Therapeutic antibodies such as trastuzumab, rituximab, and cetuximab have become central to the management of various malignancies, particularly those characterized by receptor overexpression or dysregulated signaling. However, despite their specificity, many therapeutic antibodies are associated with significant adverse effects, largely due to their inability to distinguish tumor tissues from normal tissues that express the same tumor-associated antigens at lower levels. This phenomenon, known as “on-target, off-tumor” toxicity, remains a challenge in targeted therapy, limiting both dosing and treatment duration [2–5]. To address this limitation, the concept of antibody prodrugs, engineered antibody formats that remain inactive in circulation and become reactivated specifically within the tumor microenvironment, has gained significant interest [2–5]. These constructs are designed to exploit tumor-associated features such as aberrant protease activity, low pH, hypoxia, or redox potential to trigger site-specific unmasking of the antibody paratope. Among these, protease-activated prodrugs have emerged as the most advanced approach due to the well-characterized overexpression of certain proteases, such as matriptase, cathepsins, and MMPs, in many solid tumors (Fig. 1) [5–7]. By incorporating protease-cleavable linkers and masking moieties, these designs enable spatially controlled activation of the antibody, thereby improving the therapeutic index [8]. A typical antibody prodrug design involves the fusion of a masking domain to the antibody’s antigen-binding site, physically or competitively blocking the interaction with the target antigen. Upon reaching the tumor site, protease cleavage removes the masking element, restoring binding and effector functions. A wide range of masking strategies have been explored, including sterically bulky domains, intrinsically disordered peptides, peptide–polymer conjugates, and binding domains with defined affinity [8–12]. Importantly, three protease-activated antibody prodrugs have entered clinical trials (NCT03993379; NCT03149549; NCT03369223), demonstrating the translational feasibility of this approach and underscoring the need for continued innovation. One particularly relevant antibody is cetuximab, a chimeric monoclonal IgG1 that targets the epidermal growth factor receptor (EGFR). Cetuximab is approved for use in metastatic colorectal cancer and head and neck squamous cell carcinoma [13, 14]. EGFR is overexpressed in a variety of epithelial tumors and contributes to oncogenic signaling through pathways such as MAPK, PI3K/AKT, and STAT3 [15]. By blocking ligand binding, cetuximab disrupts EGFR-mediated signaling and triggers downstream effects, including receptor internalization, G1 cell cycle arrest, and ADCC [16, 17]. However, EGFR is also abundantly expressed in normal tissues, particularly in the skin, liver and gastrointestinal tract. As a result, cetuximab therapy is frequently associated with cutaneous toxicities, affecting up to 90% of patients [18, 19]. These toxicities not only impair patient quality of life but may also necessitate dose reductions or treatment discontinuation. Given the widespread clinical use of cetuximab and its known toxicological profile, it represents an attractive candidate for prodrug conversion [20]. In this study, we sought to develop and explore a protease-activated cetuximab prodrug by introducing an anti-idiotypic affibody masking domain that selectively blocks the antigen-binding site of cetuximab until proteolytic activation. Affibody molecules (Z domains) are small (~ 6–7 kDa), engineered affinity proteins originally derived from the B domain of *Staphylococcus aureus* protein A [21, 22]. Novel affibody binders are typically generated from combinatorial libraries using display technologies such as phage, *Escherichia coli*, or *Staphylococcus carnosus* display [21, 23–30]. While affibodies have previously been used as active targeting moieties or even as intramolecular masks in affibody-drug conjugates [31–36], their application as masking domains for full-length antibody prodrugs has not been explored. To this end, we constructed a highly diverse (~ 10¹¹) affibody library displayed on the surface of *E. coli* and performed iterative rounds of magnetic and fluorescence-activated cell sorting (MACS/FACS) to identify binders targeting the paratope of cetuximab. The display system was further adapted to enable high-throughput functional screening of candidate affibodies for their masking efficiency. Top-ranked candidates were evaluated in silico using AlphaFold3 structure prediction and characterized biophysically for thermostability, refolding capacity, and binding kinetics. The most promising variant, Zcet-1, was genetically fused to cetuximab via a protease-cleavable linker and evaluated in a series of functional and biodistribution assays. The resulting prodrug displayed strong masking in vitro, reducing the functional binding capacity of cetuximab by over 400-fold. Radiolabeling and biodistribution studies further indicated that uptake of the prodrug in EGFR-positive tumors depended on the presence of active matriptase, supporting the mechanism of protease-triggered activation in vivo. Overall, these results support the potential of affibodies as versatile masking domains in antibody prodrug constructs, offering a promising approach to enhance the safety and efficacy of antibody therapies.

## Materials and methods

### Proteins and protein labelling

Cetuximab (Erbitux^®^, Apotea, Sweden) was biotinylated using Biotin-XX Microscale Protein Labelling Kit, (Invitrogen, USA) according to manufacturer’s recommendations. Human serum albumin (HSA) (Albumina Kabi 20%, Kabivitrum, Sweden) was labelled with Alexa fluor-647 following the manufacturer recommendations (Alexa Fluor™ 647 NHS Ester, Invitrogen, US). Recombinant human EGFR protein (Recombinant Human EGFR Protein, ECD, His Tag) was purchased from SinoBiologicals (China). Recombinant human matriptase (catalytic domain) was purchased from Biotechne (US). Absorbance at 280 nm was measured to determine the protein concentrations.

### Bacterial culture and library expression

*E. coli* BL21 (DE3) cells, expressing a library of 10^11^ different affibody variants [28], were grown O/N in Luria-Bertani (LB) media supplemented with carbenicillin (100 µg/mL). The next morning, the cells were diluted with fresh LB medium to an OD_600_=0.1, grown at 37 °C 150 rpm until an OD_600_=0.7, and then induced with L-arabinose (L(+)-Arabinose, 99+%, Thermo Scientific Chemicals, US) to a final concentration of 0.6% and incubated at 37 °C for 16 h.

### Library selections against cetuximab CDRs using MACS

Protein A-coated Dynabeads (Invitrogen, USA) were washed twice with PBS-P (phosphate-buffered saline supplemented with 0.1% Pluronic^®^ F108 NF Surfactant, pH 7.4; BASF Corporation, USA) and labelled with cetuximab or a non-related mix of human polyclonal IgG (IgG-mix) for 1 h at RT with gentle rotation. Afterwards, the antibody-coated magnetic beads were washed with PBS-P. In parallel, a volume of cells corresponding to 2 × 10^11^ cells were washed twice with PBS-P. First, the cells were incubated with 100 µL of pre-washed non-coated magnetic beads in a final volume of 20 mL for 30 min at RT and 15 rpm (first negative selection). The beads were captured with a magnet for 10 min and the supernatant was collected. Cells in the supernatant were next incubated with 100 µL beads coated with the non-related IgG-mix (second negative selection) in a final volume of 20 mL. The cells were incubated for 30 min at RT and 15 rpm rotation. Beads were captured with a magnet for 10 min and the supernatant was collected. Finally, the supernatant was incubated with the cetuximab-coated beads for 1 h at RT and 15 rpm rotation in a final volume of 20 mL (positive selection). The tubes were placed on a magnetic rack for 10 min to capture the beads, the supernatant was discarded, and the beads were washed with 20 mL PBS-P. Bead capture and washing was repeated three times. Lastly, the bead-bound cells were resuspended in 50 mL of LB medium supplemented with carbenicillin (100 µg/mL). The cultures were incubated O/N at 37 °C and 150 rpm. Serial dilutions of samples taken after the magnetic sorting were spread on agar plates to calculate the library size after each round. The MACS selection was repeated, adjusting the volumes to the number of cells, aiming to cover the library > 10-fold. In order to analyze the enrichment of the library outputs by flow cytometry, 10 µL of induced O/N grown libraries were washed with 200 µL of ice-cold PBS-P and incubated with 50 µL of 50 nM of biotinylated cetuximab. The samples were incubated for 1 h at RT and 150 rpm. The cells were washed twice with ice-cold PBS-P and incubated with 50 µL of 225 nM Alexa fluor 647-Human Serum Albumin conjugate (Alexa fluor 647-HSA) and 33.3 nM streptavidin R-phycoerythrin conjugate (SAPE) in PBS-P. The cells were washed again with ice-cold PBS-P and resuspended in 200 µL of ice-cold PBS-P to be analyzed in a Cytoflex S flow cytometer (Beckman Coulter, USA).

### Library selections against cetuximab CDRs using FACS

Cells from the last MACS output were induced with 0.6% arabinose as described above and collected after an overnight growth. OD_600_ was measured and a volume of cells corresponding to 10x the library size was washed twice with 800 µL of ice-cold PBS-P and treated with 100 µL of biotinylated cetuximab for 1 h at RT and 15 rpm. Initially, 150 nM of biotinylated cetuximab were used for the first sorting, followed by 50 nM for the subsequent sortings. An off-rate selection was included in the last selection round aiming to remove binders with a fast dissociation. For this, after target binding, the cells were washed and incubated in PBS-P for 3 h at 37 °C with washes every 30 min. The cells were thereafter washed again with PBS-P and incubated with 100 µL of 225 nM Alexa fluor 647-HSA and 33.3 nM SAPE in PBS-P for 30 min at 4 °C. Finally, the cells were washed twice with PBS-P and the samples were analyzed and sorted using a FACS CytoFLEX SRT instrument (Beckman Coulter, USA). Sorted cells were grown in fresh LB medium supplemented with carbenicillin (100 µg/mL) at 37 °C 150 rpm. After 16 h, the cultures were aliquoted as glycerol stocks (20% glycerol solution) and stored at -80 °C. The output libraries were analyzed by flow cytometry as described in the previous section.

### Single candidate analysis by flow cytometry

Dilutions of the library outputs were plated in agar plates supplemented with carbenicillin (100 µg/mL) and grown O/N at 37 °C. Single colony candidates were randomly picked from the agar plates and grown in LB overnight at 37 °C, 150 rpm. The cells were induced with 0.6% arabinose as described for the libraries. After O/N growth, 10 µL of each culture were washed twice with 200 µL of ice-cold PBS-P and resuspended in 20 µL of either 25 nM of biotinylated cetuximab or 25 nM biotinylated cetuximab pre-incubated for 30 min with 100 nM EGFR. After 1 h incubation at RT, the cells were washed twice with ice-cold PBS-P and labelled with 20 µL of 225 nM Alexa fluor 647-HSA conjugate and 33.3 nM streptavidin R-phycoerythrin conjugate (SAPE) in PBS-P for 30 min at 4 °C. The cells were washed twice with ice-cold PBS-P and resuspended in 100 µL of ice-cold PBS-P. The cells were analyzed using a CytoFLEX S (Beckman Coulter, USA).

### Deep sequencing of libraries

Overnight cultures from the original library and last sorting outputs were used for plasmid extraction (Qiagen Miniprep Kit, Qiagen, US). The purified plasmids were used as templates for PCR amplification using Phusion polymerase (Thermo Fisher, US) and primers containing adapter regions and Trueseq indexes (Illumina, US). The PCR products were purified using a Qiagen gel extraction kit (Qiagen, US) and the concentration was measured by Qubit (Thermo Fisher, US). The variability of the sorted libraries was analyzed by deep sequencing at the National Genomics Infrastructure (NGI, Sweden) following the manufacturer’s recommendations (Illumina, US). Between 1.8 − 0.3 million sequences were obtained from each individual sample. The libraries were annotated to match the affibody scaffold and clustered in sequence groups with 100% identity on the affibody variable regions. The outputs from the different rounds were compared to understand which sequences were more enriched throughout the selection rounds. Additionally, a pair-wise comparison was performed between the 10 most enriched affibody sequences to determine the amino acid similarity in the variable regions. The sequencing data were processed using PipeBio version 1.0.5 (PipeBio, US) and GraphPad Prism 10 (GraphPad, USA).

### In silico predictions of protein structure

The protein predictions were performed using AlphaFold3 [37] and the structures were visualized in ChimeraX [38]. The EGFR-cetuximab complex structure (PDB ID:1YY9) was downloaded from the Protein data bank [39].

### Cloning and recombinant production of affibodies

The DNA sequences of the selected affibody masking candidates were analyzed by Sanger sequencing (Microsynth, Switzerland) and cloned by In-fusion cloning (In-fusion kit, Takara, Japan) into a pET45 expression vector (Addgene, USA). *E. coli* BL21 (DE3) cells (Thermo Scientific, USA) were transformed with the sequence verified plasmids by standard heat-shock treatment and plated in agar plates supplemented with 100 µg/mL of carbenicillin. A single colony from the transformation was inoculated and grown O/N in 10 mL of Tryptic Soy Broth with yeast extract media (TSB-Y) supplemented with 100 µg/mL of carbenicillin. After 16 h, the O/N culture was diluted to OD_600_ = 0.1 in TSB-Y supplemented with 100 µg/mL of carbenicillin to a final volume of 100 mL. The culture was induced with IPTG to a final concentration of 1 mM at OD_600_ = 0.7 and incubated O/N at 25 °C and 150 rpm. After 16 h, the cells were harvested by centrifugation for 10 min at 5000 x *g*. Subsequently, the cells were lysed by sonication for 1.5 min (1s ON/1s OFF) followed by centrifugation at 4 °C and 10,000 x g for 20 min. The supernatant was filtered (0.45 μm) and the proteins were purified using immobilized metal ion affinity chromatography (IMAC). For this purpose, PD-10 columns packed with 3 mL TALON Metal Affinity Resin (TALON^®^ Superflow™ Cytiva, Sweden), wash buffer (50 mM Na_2_HPO_4_, 500 mM NaCl, 15 mM imidazole, pH 7.2) and elution buffer (50 mM Na_2_HPO_4_, 500 mM NaCl, 300 mM imidazole, pH 7.2) were used according to manufacturer’s recommendations (TALON^®^ Superflow™, GE Healthcare, Sweden). Finally, the buffer was exchanged to PBS using PD-10 desalting columns (GE Healthcare, Sweden). Purified proteins were analyzed by mass spectrometry (MS) (4800 MALDI TOF/TOF, Applied Biosystems, USA) and SDS-PAGE (NuPAGE, Invitrogen, USA). Circular dichroism spectroscopy was conducted using a Chirascan spectropolarimeter (Applied Photophysics, UK) to evaluate the protein conformation and thermostability. Thermal stability was evaluated by monitoring the change in ellipticity at 221 nm while heating the samples from 20 to 95 °C. The melting temperatures (Tm) were determined by curve fitting the variable temperature measurements (VTM) using a Boltzmann Sigmoidal model (GraphPad Prism 10, US).

### SPR analysis of masking capacity

The binding and masking abilities of the affibody molecules were investigated by surface plasmon resonance (SPR) using a Biacore 8 K instrument (GE Healthcare, Sweden). Cetuximab was immobilized on the different channels of a CM5 sensor chip (GE Healthcare, Sweden) according to the manufacturer’s recommendations. PBS-T (PBS supplemented with 0.05% Tween-20) was used as running buffer. The kinetics of the affibody candidates were analyzed by injecting a serial dilution of each candidate (37 nM to 0.05 nM in 1:3 dilutions) over cetuximab immobilized on the chip. The curves were fitted to a Langmuir 1:1 model. To investigate the competition between the masking domains and EGFR, the affibody candidates and recombinant EGFR were injected individually at a concentration of 100 nM, as well as mixed at a 1:1 ratio to assess their binding. On another CM5 sensor chip, recombinant EGFR was immobilized according to the manufacturer’s recommendation. To investigate the masking ability of the affibodies, 50 nM cetuximab was pre-incubated for 30 min at RT with 5000 nM of the respective affibody candidate, prior to injection over immobilized EGFR. The experiments were performed at 25 °C with a flow rate of 30 µL/min.

### Flow cytometry evaluation of masking domains in H292 cells

For the first experiment, a solution of 0.1 nM cetuximab was prepared and aliquots of 100 µL were mixed with the respective affibody (Zcet-1, Zcet-2, Zcet-6 or Zwt) to a final concentration of 5000 nM. A control sample was incubated with PBS-B only. The samples were incubated for 1 h at RT. For the second experiment, a serial dilution of cetuximab (250 nM to 0.004 nM in 1:3 dilutions) was prepared in PBS, and 100 µL samples of the different cetuximab concentrations were pre-incubated with 5000 nM of Zcet-1 or only PBS for 1 h. In both cases, H292 cells expressing EGFR (NCI-H292-Human lung mucoepidermoid carcinoma cells, ATCC, US) were cultured in RPMI-1640 Medium (Thermo Fisher Scientific, US) supplemented with 10% FBS (Fetal Bovine Serum, Thermo Fisher Scientific, US) at 37 °C in a 5% CO_2_ atmosphere. TrypLE™ Express Enzyme (Gibco, US) was used to detach the cells from the culture flask. The cells were harvested by centrifugation at 600 x g for 6 min and resuspended in PBS-bovine serum albumin 1% (BSA, Sigma-Aldrich, US) (PBS-B). A total of 10^5^ cells were added into each well of a 96-well plate and washed twice with 200 µL of PBS-B. The cell pellets were resuspended in 100 µL of the previously described antibody solutions. After 1 h incubation at RT, the cells were washed twice with PBS-B and resuspended in 100 µL of a 1:1000 solution of anti-human antibody labelled with Alexa fluor 488 (Goat anti-Human IgG (H + L) Cross-Adsorbed Secondary Antibody, Alexa Fluor™ 488, Invitrogen, US) in PBS-B. The cells were protected from light and incubated for 30 min at 4 °C. Finally, the cells were washed twice with PBS-B and resuspended in 100 µL of PBS-B for further analysis using a CytoFLEX S flow cytometer. One-way ANOVA was used to compare the fluorescence signal of the samples treated with cetuximab pre-incubated with different masking domains or just PBS. The fluorescence signals from the antibody serial dilutions samples were used to plot a concentration vs. response curve that was fitted using a non-linear regression model to calculate the EC_50_ (GraphPad Prism 10).

### Subcloning and recombinant production of cetuximab prodrugs

The heavy chain and light chain sequences of cetuximab were cloned into a pcDNA3 derived vector (Invitrogen, US). For the prodrug constructs, the gene encoding for the affibody domain was fused to the N-terminal of the heavy chain by a flexible linker (G_4_S) that includes a protease substrate sequence. Three linker lengths were tested, incorporating 1, 2, or 3 G_4_S repetitions surrounding the substrate sequence. A matriptase substrate [33] (LSGRSDNH) and a TEV protease substrate [35] (ENLYFQG), referred to as non-cleavable linker, were used. The cloning was performed using an infusion cloning kit following manufacturer recommendations (Takara). After verifying the plasmid sequences (Microsynth, Switzerland), O/N cultures of the transformed *E. coli* cells were used to extract the plasmids using Qiagen Midiprep kit (Qiagen, US) following the manufacturer recommendations. The plasmids were transfected into ExpiCHO cells (ExpiCHO-S™ Cells, Gibco, US) using an expifectamine transfection kit (Gibco™ ExpiFectamine™ CHO Transfection Kit), following manufacturer recommendations. After 12 days of production, the cells were harvested and the supernatants were purified by affinity chromatography using a Protein A column (HiTrap MabSelect PrismA, GE healthcare) and an ÄKTA start system (GE healthcare) following manufacturer recommendations. The solutions of purified antibodies were buffer exchanged to PBS using PD-10 desalting columns and stored at -20 °C for further characterization. The sample purity was analyzed with SDS-PAGE and size exclusion chromatography (SEC). In order to cleave off the masking domain, the prodrug was treated with 50 nM of recombinant human matriptase at 37 °C O/N and the cleavage was verified with SDS-PAGE. Thermal stability of antibody constructs was assessed using nano differential scanning fluorimetry (nanoDSF) on a Prometheus NT.48 instrument (NanoTemper Technologies). Samples were diluted to approximately 0.4 mg/mL in PBS and loaded into standard capillaries. Intrinsic tryptophan fluorescence (350/330 nm ratio) was monitored during a temperature ramp to determine melting temperatures.

### Cell binding assays by flow cytometry

For the first experiment, 100 µL solutions of the prodrugs with 1, 2 or 3 spacer linkers and cetuximab were prepared in PBS (0.5 nM final concentration). For the second experiment, the prodrug sample was treated with recombinant human matriptase as described above to cleave off the masking domain. 50 µL solutions of prodrug, cleaved prodrug and cetuximab were prepared at a range of concentrations from 500 nM to 0.0005 nM (in 1:10 dilutions). H292 cells were grown and harvested as described above. A total of 10^5^ cells were added into each well of a 96-well plate and the cells were washed twice with 200 µL PBS-B. The cell pellets were resuspended in the previously described antibody solutions. The samples were incubated for 45 min at RT with gentle shaking. The cells were washed twice with PBS-B and the pellets were resuspended in a 50 µL solution of 1:1000 anti-human antibody labelled with Alexa fluor 488 in PBS-B. The samples were incubated for 30 min at 4 °C with gentle shaking. Finally, the cells were washed twice and resuspended in 100 µL PBS-B to be analyzed by flow cytometry using a Cytoflex S instrument. One-way ANOVA was used to compare the fluorescence signals from the prodrugs with different linker lengths and cetuximab (GraphPad Prism 10). The fluorescence signals from the serial dilutions of the prodrug samples were used to plot a concentration vs. response curve that was fitted using a non-linear regression model to calculate the EC_50_ (GraphPad Prism 10).

### In vitro proliferation assays in H292 cells

H292 cells in RPMI-1640 Medium (Thermo Fisher Scientific, US) supplemented with 1% FBS were seeded in a 96-well plate (3000 cells/well). After 24 h, 100 µL of the antibody prodrugs and controls were added at a range of concentrations from 500 nM to 0.005 nM (1:10 dilutions in RPMI-1640 medium). The cells were incubated for 72 h at 37 °C and 5% C0_2_. Finally, cell viability was assessed using AlamarBlue™ cell viability reagent (ThermoFisher, US) and a CLARIOstar plate reader (BMG Labtech, Germany) following the manufacturer’s instructions. The fluorescence signal of each sample was used to plot a concentration vs. response curve. The IC_50_ values were analyzed by fitting the curves using a non-linear regression model in GraphPad Prism 10.

### Fluorescence microscopy

H292, A431, HaCaT and FaDU cells were grown according to manufacturer recommendations (ATCC) and seeded in a 96-well plate (3000 cells/well). After 16 h, the cells were washed with 200 µL sterile PBS twice and treated with 30 µL of 4% paraformaldehyde solution for cell fixation. After 20 min incubation, the paraformaldehyde was removed, and the cells were washed once with PBS and twice with PBS-B. A 100 µL solution of each antibody prodrug, at a concentration equal to the calculated cetuximab EC_50_ was used (0.1 nM) in PBS-B, was added to the cells. After 45 min incubation at RT, the cells were washed twice with PBS-B and the secondary anti-human antibody labelled with Alexa fluor 488 was added (1:1000 dilution in PBS-B). After 30 min incubation in the dark, the cells were washed with PBS twice and resuspended in PBS. The samples were observed in a fluorescence microscope (Nikon eclipse Ti, Nikon Instruments, Japan).

### Radiolabeling of antibodies

All buffers used for conjugation and radiolabeling were purified from metal contaminants using Chelex 100 resin (Bio-Rad Laboratories, USA) and filtered through a 0.22 μm filter. [^111^In]InCl₃ was obtained from Curium Pharma (Netherlands). NAP-5 columns used for purification were purchased from GE Healthcare (Sweden). Cetuximab and cetuximab prodrug were conjugated to the CHX-A’’DTPA chelator for radiolabeling with indium-111, following the previously described method [40]. Briefly, cetuximab and cetuximab prodrug (1.2 mg) were dissolved in 195 µL of 0.07 M sodium borate (pH 9.3). A freshly prepared solution of CHX-A’’DTPA (20 µL, 1 mg/mL in 0.07 M sodium borate) was added, and the mixture was incubated at 35 °C for 4 h. CHX-A’’DTPA-cetuximab and CHX-A’’DTPA-cetuximab prodrug were purified using NAP-5 columns pre-equilibrated and eluted with 0.2 M ammonium acetate (pH 5.5). The purified conjugates were stored at -20 °C until radiolabeling.

Radiolabeling was performed by adding [^111^In]InCl_3_ (40 MBq, 70–120 µL) to cetuximab or cetuximab prodrug (50 µg, 35–40 µL) in 300 µl of 0.2 M ammonium acetate (pH 5.5). The reaction mixture was vortexed and incubated at RT for 1 h, while the cetuximab prodrug was incubated for up to 2.5 h. Before purification, a 500-fold molar excess of ethylenediaminetetraacetic acid (EDTA) was added to both reactions to complex non-bound indium-111 and the reactions were incubated at RT for 10 min. Radiolabeled [^111^In]In-CHX-A’’DTPA-cetuximab and [^111^In]In-CHX-A’’DTPA-cetuximab prodrug, abbreviated as [^111^In]In-cetuximab and [^111^In]In-cetuximab prodrug, were purified using NAP-5 size-exclusion column pre-equilibrated and eluted with PBS (pH 7.4). Radiochemical yield and purity were determined using instant thin-layer chromatography (iTLC) (Agilent Technologies) eluted with 0.2 M citric acid (pH 2.0). Activity distribution along the iTLC strips was analyzed using a Storage Phosphor System (CR-35 BIO Plus, Elysia-Raytest, Germany) and AIDA Image Analysis software (Elysia-Raytest). To prevent radiolysis during storage, 1% BSA in PBS was added to each radiolabeled compound [40].

### In vitro binding specificity of radiolabeled antibodies in FaDu and H292 cells and spheroids

FaDu and H292 cells were cultured in complete RPMI-1640 medium supplemented with 10% fetal bovine serum and penicillin–streptomycin at 37 °C and 5% CO_2_. When the cells reached 80–90% confluency, they were collected using a 0.25% EDTA-trypsin solution. Binding specificity and internalization assays were performed as described earlier [40].

Briefly, for the binding specificity assay, the cells were seeded in 6-well plates (1 × 10⁶ cells/well) the day before the experiment. The cells in the blocked groups were pre-incubated with non-labeled cetuximab (100 nM) for 30 min, while the cells in the non-blocked groups were incubated with media. Then, [^111^In]In-cetuximab or [^111^In]In-cetuximab prodrug (1 nM) were added and the cells were further incubated for 1 h at 37 °C. Samples containing media and cells were collected and measured using an automated gamma counter (Wizard 2480, PerkinElmer, USA). Cell-associated activity was calculated as percentage from the total added activity. To study internalization, the cells were incubated with [^111^In]In-cetuximab (1 nM) for 1, 2, 4, 6, and 24 h at 37 °C. At every time point, three fractions were collected: media, membrane-bound activity (using acid wash method) and internalized activity, the activity was measured using an automated gamma counter. Cell-associated activity was calculated as percentage from the total added activity. The data were normalized to the highest value of cell-associated activity at 24 h taken as 100%. For preparation of spheroids, plastic molds designed for 81-well spheroid formation were used. They were prepared from FormLabs Gray Resin 1 L photopolymer resin (USA) using a FormLabs Form3 3D printer (USA). A 1.5% agarose solution in sterile PBS was heated to boiling and poured into the molds. After solidification, the agarose molds were carefully removed and placed in 12-well plates. To form spheroids, 190 µL of a cell suspension (1.7 × 10⁶ cells/mL, 4000 cells per well) was added to each agarose well. The plates were incubated at room temperature for 30 min to allow cell sedimentation, followed by the addition of 2 mL of complete RPMI medium. Spheroids were maintained for three days in a CO₂ incubator (37 °C, 5% CO₂) before experiments. To measure the binding of [^111^In]In-cetuximab and [^111^In]In-cetuximab prodrug to spheroids, the spheroids were collected by gentle washing with media, transferred to non-adhesive 24-well plates, and counted. To block EGFR binding, a group of spheroids was pre-incubated with 100-fold molar excess of non-radiolabeled cetuximab (1 µM) for 30 min at 37 °C (blocked group). Then, spheroids were incubated with [^111^In]In-cetuximab or [^111^In]In-cetuximab prodrug (10 nM) for 24 h (non-blocked group). After incubation, spheroids were transferred to 1.5 mL low-adhesion Eppendorf tubes, centrifuged at 3000 × g for 3 min, and washed three times with 1 mL of PBS. Residual media was removed, and activity was measured using a Wizard 2480 automatic gamma counter (PerkinElmer, USA). Background signal from an empty Eppendorf tube centrifuged with the same protein solution was subtracted from the measurements. The activity bound to spheroids was calculated as a percentage of the total added activity and normalized per spheroid number. To account for variations in the amount of available EGF receptors, data were further normalized to the binding of [^111^In]In-cetuximab in the non-blocked group for FaDu and H292 spheroids separately. Welch’s t-test was used for statistical analysis (GraphPad Prism 10).

### Tumor implantation and biodistribution of [^111^In]In-cetuximab and [^111^In]In-cetuximab prodrug in Balb/c nu/nu mice bearing H292 or FaDu xenografts

The animal experiments were approved by the Local Ethics Committee for Animal Research in Uppsala (permit 5.8.18–00473/2021). Female Balb/c nu/nu mice were supplied from Scanbur A/S (Karlslunde, Denmark) and had an adaptation period of one week before the start of experiments. For tumor implantation, mice were subcutaneously injected with 10 × 10^6^ H292 cells in 100 µL of culture medium with Matrigel (1:1) or with 5 × 10^6^ FaDu cells in 100 µL of culture medium in the hind leg. The experiments started seven days after the implantation. The average animal weight was 18.6 ± 1.7 g in the H292 groups, 18.5 ± 1.5 g in the FaDu groups. The average tumor weight was 0.27 ± 0.15 g for H292 xenografts, 0.36 ± 0.14 g for FaDu xenografts. To evaluate the biodistribution of [^111^In]In-cetuximab and [^111^In]In-cetuximab prodrug, mice were randomized into groups (*n* = 4) and were intravenously (i.v.) injected with [^111^In]In-cetuximab or [^111^In]In-cetuximab prodrug. Both compounds were administered at equal activities (60 kBq in 100 µL 1% BSA in PBS per mouse) and equimolar amounts (2 pmol). Tissues, organs and tumors were collected 72 h post-injection (p.i.), weighed, and measured for activity as described above. The uptake was calculated as percentage of injected dose per gram of sample weight (%ID/g). To account for variations in the amount of available EGF receptors, data were normalized to the uptake of [^111^In]In-cetuximab in H292 and FaDu tumors. Welch’s t-test was used for statistical analysis (GraphPad Prism 10).

### Other tools

Schematic figures were created using BioRender (BioRender.com) and GraphPad Prism 10 (GraphPad, USA) was used to plot and analyze data. AlphaFold 3 was used to predict the affibody protein structures and interactions with cetuximab. UCSF ChimeraX program was used to visualize the protein structure.

## Results

**Fig. 2:**
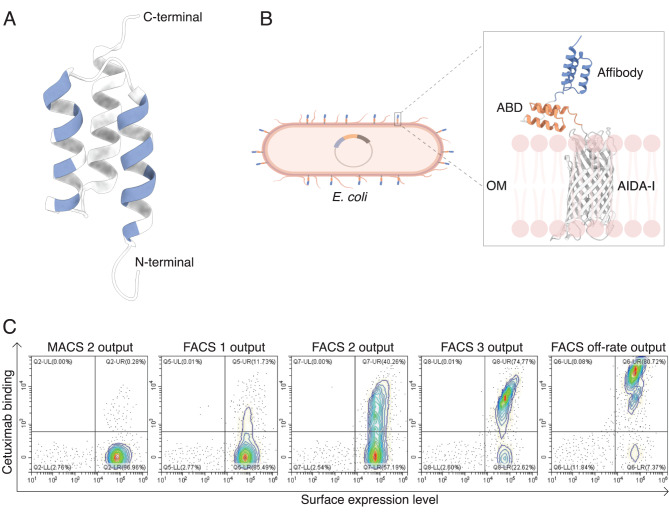
Affibody library enrichment by *E. coli* display. (**A**) Affibody protein with the different variable positions highlighted in blue. (**B**) Schematic representation of an *E. coli* cell displaying an affibody variant fused to an ABD. The displayed fusion protein is anchored to the cell membrane by an AIDA autotransporter (grey). *OM: outer membrane. *(**C**) Library selection outputs analyzed by flow cytometry after the corresponding FACS sorting. The x-axis represents the expression level (binding of HSA), while the y-axis represents cetuximab binding. Each plot corresponds to a different round of selection, illustrating the enrichment of affibody candidates with increased binding to cetuximab

### Selection of cetuximab binders by MACS and FACS

A library of 10^11^ affibody variants (Fig. 2A) was displayed on *Escherichia coli* cells to select affibody molecules with binding capacity to the complementarity-determining regions (CDRs) of cetuximab. Each cell displays several copies of a library variant fused to an albumin binding domain (ABD) [41] which is used for normalization of the surface expression during fluorescence-activated cell sorting (FACS) (Fig. 2B) [26, 28]. The library was first enriched for cetuximab binders by magnetic-assisted cell sorting (MACS) using cetuximab-coated magnetic beads. With the aim to deplete affibodies binding to the constant regions of antibodies or protein A, negative selections were performed using beads coated with a mix of unrelated IgGs and non-coated beads, respectively. The MACS procedure was repeated twice to remove the bulk of non-binders and decrease the library size. By the second MACS round, the maximum diversity of the library had decreased to about 10^5^ variants. Following MACS, three rounds of FACS were performed by incubating the cell displayed library with biotinylated cetuximab, and subsequently labelling the cells with two fluorophores to detect both surface expression and cetuximab binding (Fig. 2C). The concentration of cetuximab was reduced to increase the stringency of the selections. In the final FACS round, a 3-hour incubation at 37 °C was included following target binding to enrich for clones with slower dissociation rates (off-rate selection). Flow cytometry analysis of the sorting outputs demonstrated a clear enrichment of cetuximab-binding clones over the successive rounds (Fig. 2C).

**Fig. 3:**
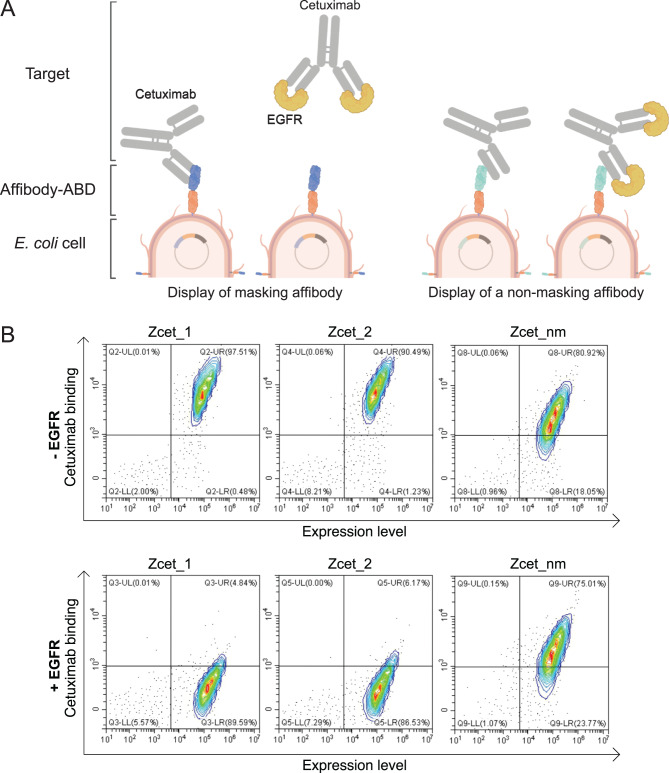
*E. coli* display method to characterize single candidates in a high-throughput manner. (**A**) Schematic representation of the *E. coli* display system strategy used to characterize the masking capacity of individual candidate affibodies. The affibodies are screened for their ability to bind cetuximab alone or pre-incubated with EGFR. While masking affibody candidates are not expected to bind cetuximab when pre-incubated with EGFR, non-masking candidates can bind cetuximab regardless of the presence of EGFR. (**B**) Flow cytometry evaluation of the selected masking candidates Zcet_1, Zcet_2 and Zcet_nm when analyzing the affibody interaction with cetuximab (-EGFR) or cetuximab pre-incubated with EGFR (+ EGFR)

### Analysis of single candidates by flow cytometry

*E. coli* clones from the FACS outputs were randomly picked and assessed individually for their binding and masking capacity to cetuximab. Cells displaying the respective candidates were labelled in two conditions: either with cetuximab alone or with cetuximab pre-incubated with EGFR (Fig. 3A). This assay enabled a high-throughput evaluation of each candidate’s ability to block the EGFR-binding site on cetuximab (Figure S1). The results showed that most candidates bound to cetuximab and competed with EGFR for an overlapping epitope (Figure S1). Figure 3B highlights the two most enriched affibody candidates, Zcet_1 and Zcet_2, both displaying strong masking capacity. A non-masking affibody (Zcet-nm) is included for comparison.

**Fig. 4:**
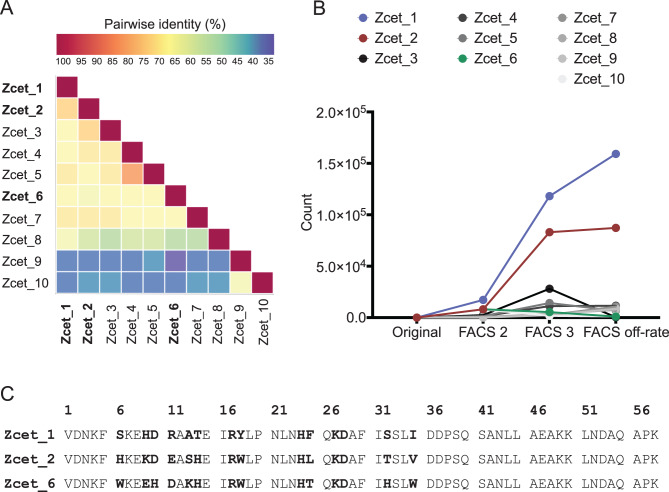
Analysis of selected affibody molecules by deep sequencing. (**A**) Pair-wise heat map indicating the identity between variable positions of the top 10 most enriched sequences. (**B**) Count of the ten most enriched variants across the selection rounds. The three selected candidates are highlighted in blue (Zcet_1), red (Zcet_2) and green (Zcet_6). (**C**) Amino acid sequence of the selected candidates

### Deep sequencing of library outputs

The original library along with the outputs from FACS rounds 2, 3, and the off-rate selection were analyzed by deep sequencing to determine the sequence distribution among the clones. Pairwise sequence similarity among the ten most enriched variants is visualized in the heat map in Fig. 4A. The top seven sequences shared more than 70% similarity in their variable positions. Analysis of sequence frequencies across the selection rounds revealed a clear enrichment of Zcet_1 and Zcet_2, which accounted for 41.8% and 22.8% of the final sorted library, respectively. These two candidates were selected for further analysis, along with a less abundant variant, Zcet_6 (0.3%), for comparison (Fig. 4B). The amino acid sequences of these affibody candidates are shown in Fig. 4C.

**Fig. 5:**
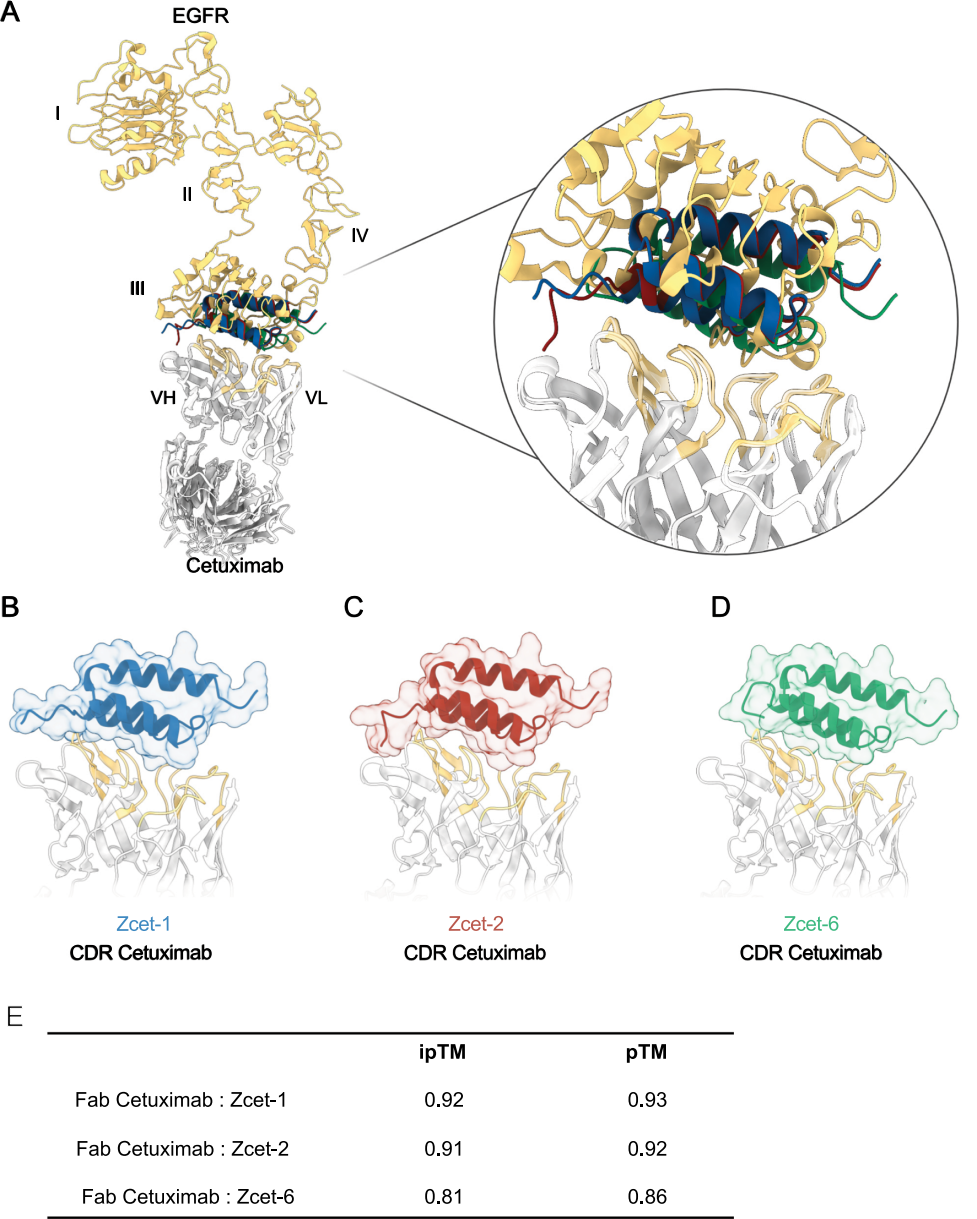
Protein structure predictions by AlphaFold3. (**A**) Superimposition of the predicted affibody-Fab cetuximab complexes with the crystal structure of EGFR – Fab cetuximab (PDB ID: 1YY9) [32]. The interactions are predicted to occur in the CDR loops, which are highlighted in yellow. (**B-E**) AlphaFold models showing the interaction between the respective affibody masking domain and cetuximab Fab. (**E**) Zcet-1 and Fab cetuximab complex (iPTM: 0.92, pTM: 0.93); Zcet-2 and Fab cetuximab complex (iPTM: 0.91, pTM: 0.92); Zcet-6 and Fab cetuximab complex (iPTM: 0.81, pTM: 0.86)

### Model of the complex structures

Structure predictions of the interactions between the selected masking domains (Zcet-1, Zcet-2, and Zcet-6) and the variable region of the cetuximab Fab were performed using AlphaFold3 [28]. The predicted models suggested that all three affibodies compete with EGFR for the same epitope on cetuximab, located within its complementarity-determining regions (CDRs) (Fig. 5A–D). Superposition of the AlphaFold3-predicted affibody–cetuximab complexes with the published EGFR–cetuximab crystal structure (PDB: 1YY9) further indicated that the predicted affibody binding interface partially overlaps with the residues involved in EGFR recognition, providing a structural rationale for the observed masking efficiency. Confidence metrics for these models are described in Fig. 5. The predicted TM-scores (pTM), all above 0.86, indicate high overall confidence in the structural models, while the interface pTM scores (ipTM), ranging from 0.81 to 0.92, support reliable predictions of the relative orientation between the affibody and cetuximab subunits. The per-residue plDDT scores are shown in Figure S2. Collectively, these models provide insight into how the affibodies may mask the cetuximab CDRs and thereby prevent EGFR binding.

**Fig. 6:**
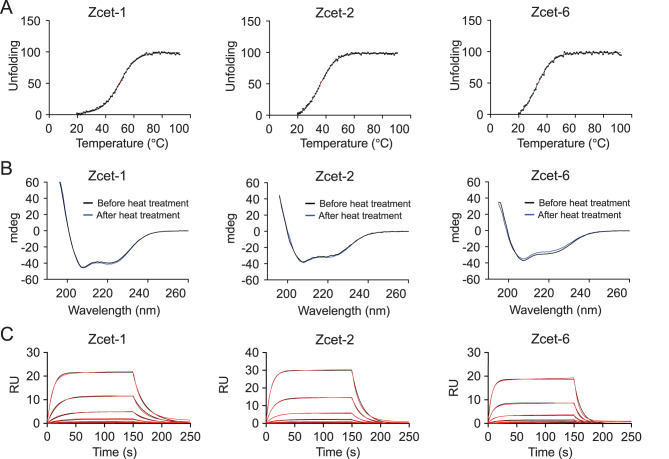
Analysis of secondary structure, thermostability and binding kinetics of affibody molecules Zcet-1, Zcet-2 and Zcet-6. (**A**) Variable temperature measurement using CD spectroscopy. (**B**) CD spectroscopy for analysis of secondary structure content. The analysis shows overlay of spectra from before and after heat denaturation, for assessment of refolding. (**C**) SPR sensorgrams showing the kinetics of binding between affibody molecules (analyte) and cetuximab (ligand). The sensorgram is shown in red, and the curve fitting in black. Affibody concentrations range from 0.049 nM to 37 nM

### Analysis of secondary structure, thermal stability and refolding

The three masking domains were produced as soluble proteins and purified, followed by characterization of their secondary structure content, thermostability, and refolding capacity. Variable temperature circular dichroism (CD) spectroscopy revealed thermal melting points (T_m_) of 50 °C for Zcet-1, 36 °C for Zcet-2, and 32 °C for Zcet-6 (Fig. 6A). Furthermore, CD spectra confirmed the expected alpha-helical structure of the affibodies and demonstrated their ability to refold after thermal denaturation (Fig. 6B).

**Fig. 7:**
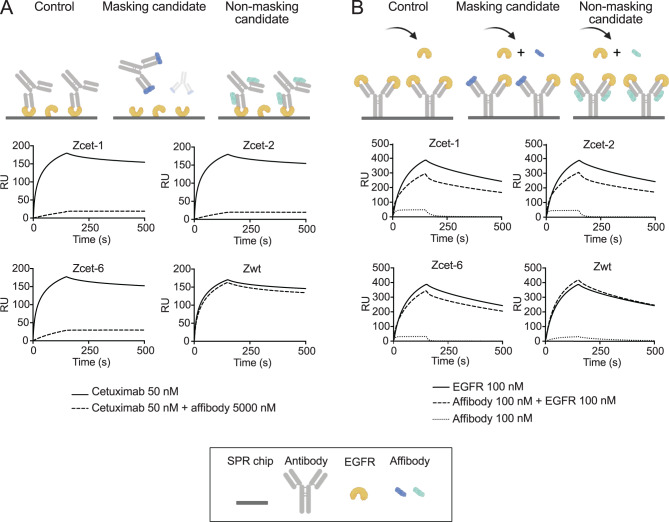
Assessment of the masking capacity of affibodies. (**A**) Blocking experiment showing the decrease of cetuximab-EGFR binding signal when cetuximab was pre-incubated with excess of masking affibody domains (Zcet-1, Zcet-2 or Zcet-6). (**B**) Competition experiment confirming that both EGFR and the masking affibody domains compete for the same epitope in cetuximab’s CDR. In both experiments, Zwt affibody, with binding capacity to the Fc region of antibodies, was used as a negative control

### Analysis of binding and masking capacity by SPR

Surface plasmon resonance (SPR) was used to study the kinetics of the interaction between cetuximab and the affibody molecules (Zcet-1, Zcet-2, and Zcet-6). Cetuximab was immobilized on an SPR chip surface, and a concentration series of each affibody was injected over the surface as analytes (Fig. 6C). The results showed similar kinetic profiles for all three affibodies, with relatively fast association and dissociation rates, corresponding to equilibrium dissociation constants (K_D_) ranging from 26.3 nM to 46.3 nM (Table 1). SPR was also used to assess the masking capacity of the three affibody candidates alongside a non-masking control, the Fc-binding affibody Zwt. In a blocking experiment, cetuximab was pre-incubated with either excess affibody (1:100 molar ratio) or buffer (control), and then injected over surface-immobilized EGFR (Fig. 7A). The results showed a substantial decrease in binding signal for cetuximab pre-incubated with the masking affibodies compared to cetuximab alone, strongly supporting that these affibodies bind within the CDRs of cetuximab and prevent EGFR binding. As expected, the Fc-binding control affibody (Zwt) had no effect on EGFR binding, confirming its non-overlapping binding epitope. In a complementary SPR assay, each affibody was pre-incubated with EGFR at a 1:1 molar ratio and the mixture injected over surface-immobilized cetuximab (Fig. 7B). The signal for the mixtures was reduced compared to EGFR alone, indicating that the affibody molecules compete with EGFR for binding to cetuximab. In contrast, co-injection of EGFR with Zwt resulted in minimal signal reduction, consistent with non-competing binding sites and simultaneous interaction. It is important to note that differences in response units (RU) also reflect the substantial difference in molecular weight between recombinant EGFR (~ 70 kDa) and the affibodies (~ 7.5 kDa). Overall, these results demonstrate that all three affibody molecules, Zcet-1, Zcet-2, and Zcet-6, effectively blocked the interaction between cetuximab and EGFR. Zcet-6 appeared to be the least efficient masking domain, consistent with its lower enrichment during FACS selection. In contrast, Zcet-1 consistently emerged as the most effective candidate in both assays.

**Fig. 8:**
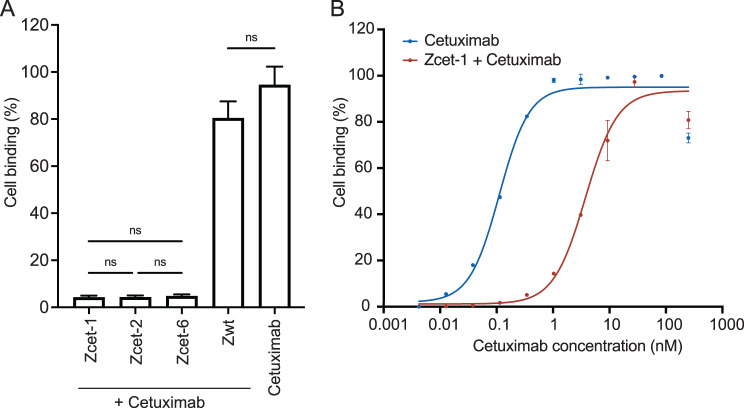
Evaluation of the affibodies masking capacity by flow cytometry. (**A**) Results from flow cytometry showing cetuximab binding to H292 cells on the y-axis. Cells were treated with cetuximab alone or cetuximab preincubated with the respective affibody molecule. The masking capacity of the affibody domains (Zcet-1, Zcet-2, Zcet-6 and control Zwt) was evaluated (*n = 2*). The difference between samples preincubated with the masking domains (Zcet-1, Zcet-2 and Zcet-6) and cetuximab control was significant (p-values = < 0.0001), while the difference between cetuximab preincubated with Zwt and cetuximab was non-significant (p-value = 0.23). The differences between the different masking domains were non-significant (p-value = > 0.99). (**B**) Dose-response evaluation by flow cytometry of cetuximab binding to H292 cells in the presence and absence of Zcet-1 (*n = 2*). The antibody concentration ranged from 0.004 nM to 250 nM, and the Zcet-1 concentration was constant (5000 nM). EC_50_ values were determined by fitting the data

### Analysis of cell binding and masking

The effects of the three masking domains on cetuximab function were further evaluated using H292 cells, which are derived from a mucoepidermoid pulmonary carcinoma and overexpress EGFR. Cetuximab antibodies were pre-incubated with an excess of affibody molecules (Zcet-1, Zcet-2, Zcet-6, or control Zwt), and binding to H292 cells was measured by flow cytometry (Fig. 8A). The results showed that the masking domains effectively inhibited cetuximab binding to H292 cells, whereas Zwt had no impact, confirming its non-masking nature. Next, a titration of cetuximab was performed with or without pre-incubation with 5000 nM of Zcet-1 or PBS (control), and binding to H292 cells was again measured using flow cytometry. The EC50 was calculated to be 0.11 ± 0.0001 nM for cetuximab alone, and 3.8 ± 0.3 nM for cetuximab pre-incubated with Zcet-1 (Fig. 8B), corresponding to a 34-fold increase in EC50, indicating substantial masking of cetuximab activity by soluble affibodies.

**Table 1:** Characterization of masking domains by SPR. Results of the kinetic analysis interaction between cetuximab and affibody masking domains (Zcet-1, Zcet-2 and Zcet-6) by SPR at 25 °C (*n* = 3)

	k_a_ (1/Ms)	k_d_ (1/s)	K_D_ (nM)
Zcet-1	3,88E + 06	1,02E-01	26,3 ± 1,2
Zcet-2	5,15E + 06	1,83E-01	36,5 ± 3,2
Zcet-6	3,77E + 06	1,93E-01	46,3 ± 8,6

### Cetuximab prodrug production and characterization

**Fig. 9:**
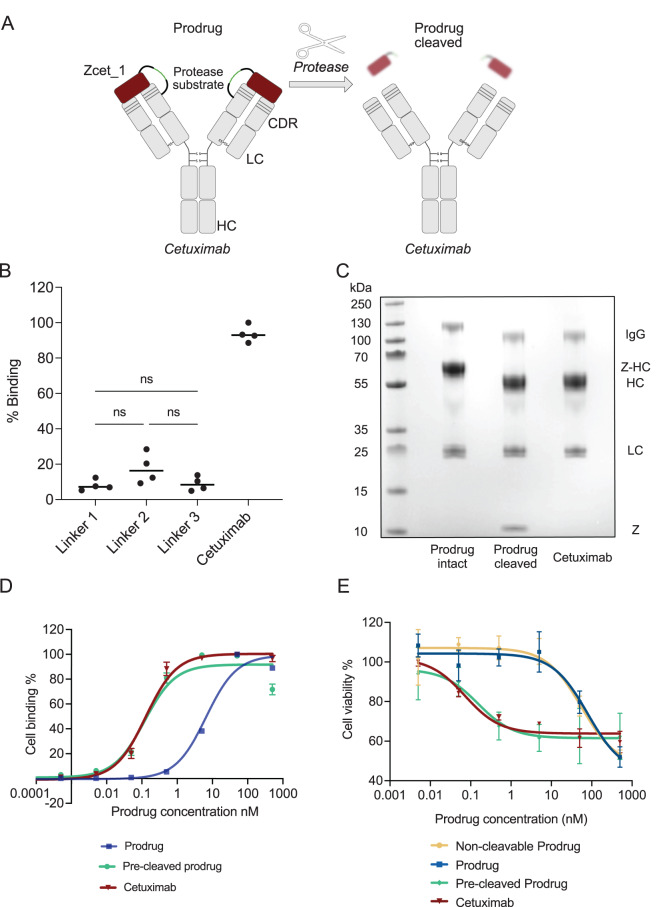
Prodrug evaluation in H292 cells. (**A**) Schematic representation of the cetuximab prodrug. The linkers contain a protease substrate and connect the affibody masking domains to the antibody. Treatment of the antibody prodrug with protease releases the masking domain. (**B**) Results from flow cytometry showing cetuximab and prodrugs with different linker lengths binding to H292 cells (*n = 4*). The differences in binding capacity between the different prodrugs were not significant (Linker 1 vs. Linker 2 p-value = 0.12, Linker 1 vs. Linker 3 p-value = 0.99, Linker 2 vs. Linker 3 p-value = 0.17). The differences between the different prodrugs and cetuximab were significant (p-value = < 0.0001) (**C**) SDS-PAGE of the purified intact prodrug with a matriptase substrate, cleaved prodrug and cetuximab as control. The different bands correspond to full size antibody or prodrug (IgG, 164 kDa), heavy chain with masking domain (Z-HC, 59 kDa), heavy chain (HC, 51.5 kDa), light chain (LC, 23 kDa) and masking domain (Z, 7.5 kDa). (**D**) Results from flow cytometry showing binding to H292 cells. Analysis of the binding capacity of intact prodrug, matriptase cleaved prodrug and cetuximab (*n = 3*). (**E**) Cell growth inhibition assay in H292 cells (*n = 3*). The EC_50_ and IC_50_ values were determined by fitting the curves

The candidate Zcet-1 was chosen as the masking domain for the design of a prototype cetuximab prodrug. The affibody was genetically fused to the N-terminus of cetuximab’s heavy chain, placing it in close proximity to the CDR loops (Fig. 9A). The linker included a protease-cleavable substrate flanked by G4S repeats. We also evaluated whether linker length influences the ability of the affibody domain to access and mask the cetuximab paratope. Based on AlphaFold3 models of the affibody–cetuximab complex (Fig. 5 and Supplementary Figure S2), the masking domain is predicted to bind directly within the CDR region of the antibody. The linker therefore primarily needs to allow the affibody domain to reach the Fab variable region while remaining tethered to the heavy-chain N-terminus. Given the relatively small size of the affibody (~ 7 kDa) and the short distance between the N-terminus of the heavy chain and the Fab paratope, relatively short flexible linkers were expected to be sufficient to enable productive masking. Three variants were therefore generated containing one, two, or three G4S repeats flanking a non-cleavable substrate and produced in ExpiCHO cells. Following purification, the yield of the prodrugs was comparable to that of unmodified cetuximab (0.5–2 mg per 10 mL culture), suggesting that fusion of the affibody domain did not markedly affect antibody expression or recovery. Size exclusion chromatography confirmed that all purified constructs were mostly monomeric, with no direct signs of aggregation (Figure S3). The ability of the prodrugs to mask cetuximab binding was evaluated in EGFR-expressing H292 cells using flow cytometry. All three linker designs (with 1, 2, or 3 G4S repeats) demonstrated efficient masking of EGFR binding, with no significant differences in performance observed between the different linker lengths (Fig. 9B). The construct with the longest linker (3 G4S repeats) was selected for further studies and engineered to contain a matriptase-cleavable substrate [33]. NanoDSF analysis revealed similar thermal unfolding profiles for the prodrug construct and unmodified cetuximab (Figure S4). The observed melting temperatures were comparable, indicating that fusion of the affibody-based masking domains did not substantially affect antibody stability (Figure S4). Upon incubation with recombinant matriptase, SDS-PAGE analysis confirmed efficient proteolytic cleavage of the linker and release of the masking domain (Fig. 9C). The binding capacity of both cleaved and intact prodrug was then assessed in H292 cells and compared to cetuximab. EC50 values were calculated as 0.13 ± 0.02 nM (cetuximab), 0.12 ± 0.01 nM (prodrug, cleaved), and 6.60 ± 0.74 nM (prodrug, intact), corresponding to a 55-fold increase in EC50 for the intact prodrug (Fig. 9D). These results confirm that the masking domain substantially reduces target binding, and that protease treatment restores binding activity to levels equivalent to the unmodified antibody, demonstrating efficient recovery of cetuximab function following proteolytic activation.

### Cell growth inhibition assay

To assess the impact of the cetuximab prodrug on the growth of H292 cells, a cell viability assay was conducted. H292 cells were incubated with various concentrations of cetuximab, intact prodrug, cleaved prodrug, and a control prodrug containing a non-matriptase-cleavable substrate, referred to as the non-cleavable prodrug control (Fig. 9E). After 72 h, the results demonstrated substantially reduced growth inhibition by the intact prodrug compared to both the pre-cleaved prodrug and cetuximab. Furthermore, the pre-cleaved prodrug showed an IC50 value comparable to the cetuximab control, indicating that the prodrug can be functionally activated by matriptase cleavage (Fig. 9E). The IC50 values were calculated as 79.3 ± 14 nM (intact prodrug), 59.9 ± 17 nM (non-cleavable prodrug), 0.08 ± 0.03 nM (cetuximab), and 0.19 ± 0.12 nM (cleaved prodrug), corresponding to a more than 400-fold increase in IC50 for the intact prodrug compared to the cleaved version.

**Fig. 10:**
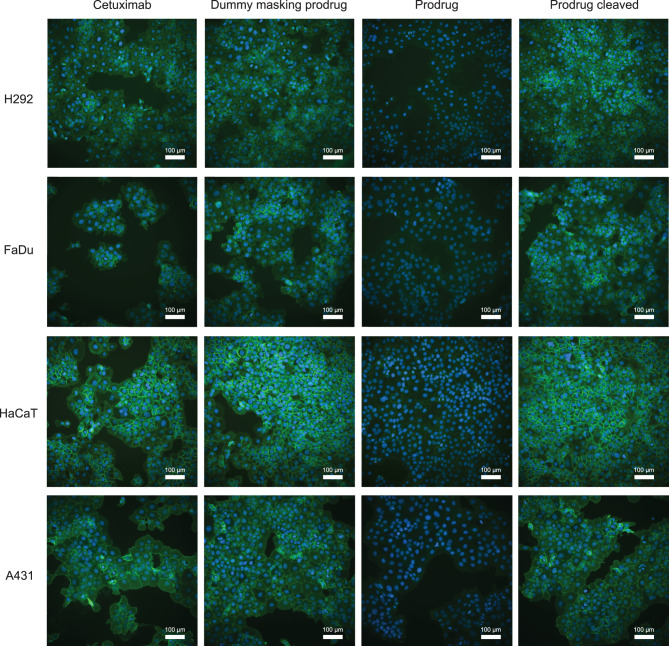
Microscopy results of cetuximab prodrug tested in H292, FaDu, HaCaT and A431 cells. The prodrug was analyzed with cell lines expressing different levels of EGFR. Cetuximab and a prodrug having a non-masking domain (Dummy masking prodrug) were included as controls. EGFR is shown in green (Alexa Fluor 488) and the nuclei are labelled in blue (DAPI). Scale bars are indicated in the image

### Microscopy

The prodrug was further analyzed in four different EGFR-expressing cell lines using fluorescence microscopy (Fig. 10). The panel included H292 cells; FaDu cells, derived from a hypopharyngeal squamous cell carcinoma; HaCaT cells, derived from immortalized keratinocytes; and A431 cells, derived from an epidermoid carcinoma. HaCaT and A431 cells are reported to express higher levels of EGFR compared to H292 and FaDu [42]. The parental antibody cetuximab was included as a reference, along with a control prodrug construct in which a non-specific affibody was fused to cetuximab (referred to as the Dummy masking control). This control was used to evaluate the potential influence of steric hindrance on masking efficacy. The results showed that the specific masking domain effectively blocked binding of the cetuximab prodrug to EGFR across all four cell lines, and that EGFR binding was restored following protease treatment (Fig. 10). Notably, the Dummy masking control exhibited minimal effect on cetuximab binding, highlighting the importance of specific interaction between the affibody domain and cetuximab for efficient masking.

### Radiolabeling, specificity assay in cells and spheroids, and biodistribution of [^111^In]In-cetuximab and [^111^In]In-cetuximab prodrug in Balb/c nu/nu mice bearing H292 or FaDu xenografts

**Fig. 11:**
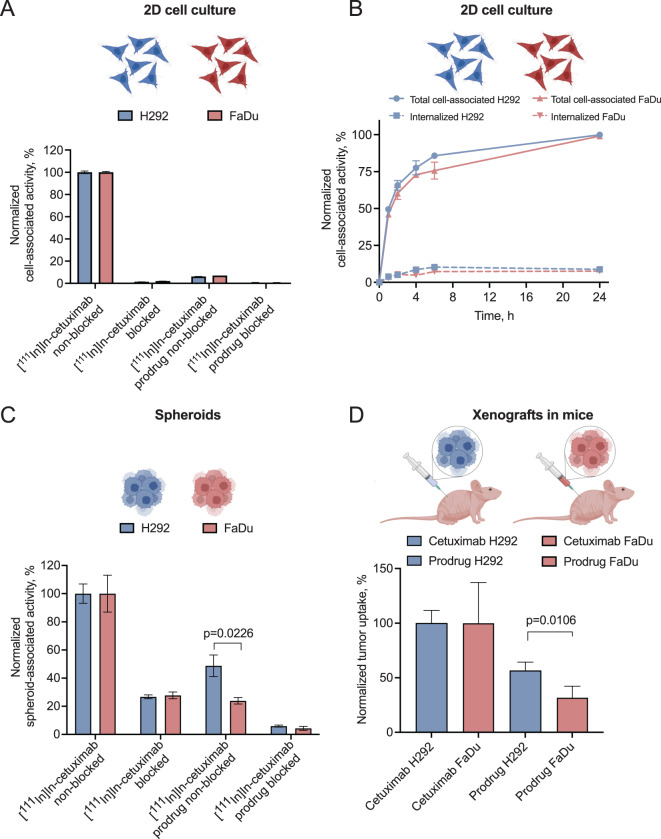
Evaluation of radiolabeled [^111^In]In-cetuximab and [^111^In]In-cetuximab prodrug in H292 and FaDu cells, spheroids, and mice bearing H292 and FaDu xenografts. (**A**) Binding specificity of [^111^In]In-cetuximab and [^111^In]In-cetuximab prodrug, the cells in the blocked groups were pre-incubated with an excess of non-labeled cetuximab (*n* = 3). (**B**) Internalization of [^111^In]In-cetuximab over 24 h incubation with the cells. (**C**) [^111^In]In-cetuximab and [^111^In]In-cetuximab prodrug activity associated to H292 and FaDu spheroids after 24 h of co-incubation (*n* = 3). (**D**) Tumor uptake of [^111^In]In-cetuximab and [^111^In]In-cetuximab prodrug in Balb/c nu/nu mice bearing H292 and FaDu xenografts, 72 h after injection (*n* = 4). Values are presented as mean ± SD

Cetuximab and the intact cetuximab prodrug were radiolabeled with indium-111 and purified to > 96% radiochemical purity (SI Table 1). A binding specificity assay was performed for both compounds using H292 and FaDu cells in monolayer culture and spheroid models. In both formats, pre-incubation with excess non-labeled cetuximab significantly (*p* < 0.01, Welch’s t-test) reduced cell-associated activity of [¹¹¹In]In-cetuximab, confirming EGFR-specific binding (Fig. 11A, C). In monolayers, the cell-associated activity of [¹¹¹In]In-cetuximab prodrug was approximately 17-fold lower than that of [¹¹¹In]In-cetuximab, indicating efficient masking of EGFR binding. No major differences in [¹¹¹In]In-cetuximab prodrug binding were observed between H292 and FaDu cells under monolayer conditions. However, in spheroids, 24-hour incubation with [¹¹¹In]In-cetuximab prodrug resulted in a two-fold higher cell-associated activity in H292 spheroids compared to FaDu, suggesting prodrug activation in the H292 model. Cellular processing of [¹¹¹In]In-cetuximab in both H292 and FaDu cells showed a similar pattern, characterized by slow internalization, with approximately 10% of the total cell-associated activity internalized after 24 h (Fig. 11B). To investigate EGFR masking and activation of [¹¹¹In]In-cetuximab prodrug in vivo, a biodistribution study was conducted comparing [¹¹¹In]In-cetuximab and [¹¹¹In]In-cetuximab prodrug in mice bearing H292 or FaDu xenografts at 72 h post-injection (p.i.) (SI Table 2). For each tumor model, [¹¹¹In]In-cetuximab uptake served as a reference to control for potential differences in EGFR expression, vascularization, perfusion, and diffusion. The uptake of [¹¹¹In]In-cetuximab prodrug was significantly higher in H292 xenografts (57 ± 8%) compared to FaDu xenografts (32 ± 11%) (*p* = 0.0106, Welch’s t-test) (Fig. 11D), supporting in vivo activation of the prodrug in the H292 model, likely due to elevated matriptase activity. [¹¹¹In]In-cetuximab had higher tumor-to-organ ratios due to higher tumor uptake and lower activity in blood, because it was taken up to a high degree by EGFR-expressing tumors, leaving a lower fraction in blood circulation (SI Table 3). The tumor-to-organ ratios for [¹¹¹In]In-cetuximab prodrug were lower, due to lower tumor uptake and higher activity in blood, reflecting a lower degree of EGFR-driven uptake.

## Discussion

Antibody prodrugs have emerged as a promising strategy to reduce on-target, off-tumor toxicities, thereby enhancing drug efficacy. In this study, we present an antibody prodrug format that employs affibody molecules as masking domains to reversibly block antibody binding, thus controlling therapeutic activity until proteolytic activation in the tumor microenvironment. We used a combinatorial affibody library displayed on *E. coli* to identify binders targeting the paratope of cetuximab. Following MACS and four rounds of FACS, we enriched for clones with desirable masking properties (Fig. 2). Individual affibody candidates were screened using a cell surface display assay to assess their masking efficiency (Fig. 3, Figure S1). This workflow demonstrates the utility of the *E. coli* display platform not only for high-throughput selection of candidate binders, but also for direct functional assessment of masking capacity. Most candidates bound to cetuximab and exhibited some degree of masking capacity. Deep sequencing of enriched populations identified Zcet-1 and Zcet-2 as top candidates and three affibodies (Zcet-1, Zcet-2, and Zcet-6) were selected for further characterization. AlphaFold3 structural predictions suggested that all three binders target a similar epitope within the CDR loops of cetuximab [43], overlapping with its EGFR-binding site (Fig. 5). Circular dichroism analyses confirmed that all three candidates retained the expected alpha-helical structure and demonstrated reversible thermal unfolding, with Zcet-1 exhibiting the highest thermal stability (Fig. 6). This stability likely contributed to its enrichment under off-rate selection conditions at 37 °C.

It has been proposed that masking domains suitable for prodrug applications should exhibit intermediate affinity with relatively rapid off-rates, ensuring efficient inhibition of antibody–antigen binding while still permitting quick release and reactivation after proteolytic cleavage [44]. The binding kinetic analysis showed that all three affibody candidates exhibited fast association and dissociation rates, consistent with these criteria. Despite their relatively rapid off-rates, both surface plasmon resonance (SPR) and flow cytometry confirmed that the affibody domains effectively blocked cetuximab binding to EGFR (Figs. 7 and 8), demonstrating their potential as reversible and efficient masking elements in antibody prodrug design.

Encouraged by these findings, we constructed a cetuximab prodrug by genetically fusing Zcet-1 to the N-terminus of the heavy chain via a protease-cleavable linker (Fig. 9). A panel of constructs with varying linker lengths showed efficient masking in all configurations, with no major differences observed. Size-exclusion chromatography revealed profiles similar to those of non-modified cetuximab, indicating that the prodrugs were predominantly monomeric. Moreover, nanoDSF analysis showed similar melting temperatures for the prodrugs compared with cetuximab, suggesting that genetic fusion of the masking domains did not substantially affect antibody stability. These results are important, as reduced solubility or stability could negatively impact future clinical performance, for example by promoting aggregation or immunogenicity. We selected the longest linker variant for further development and introduced a cleavage site for matriptase, a tumor-associated protease previously used for prodrug activation [20, 45]. The resulting prodrug showed a 55-fold increase in EC50 for binding and a > 400-fold increase in IC50 for cell growth inhibition compared to cetuximab in EGFR-positive H292 cells (Fig. 9), corresponding to a masking factor (MF) of approximately 417. Importantly, activation by recombinant matriptase restored growth inhibition to levels comparable to cetuximab. In previous work, we demonstrated by western blot analysis that the H292 cell line expresses matriptase in a form not complexed with its endogenous inhibitor hepatocyte growth factor activator inhibitor-1 (HAI-1) suggesting enzymatic activity [31]. In the same study, expression of urokinase-type plasminogen activator (uPA) was also confirmed in H292 cells [31]. However, in a subsequent substrate-specific analysis we showed that the LSGRSDNH peptide substrate is negligibly cleaved by uPA [33]. In the same study, cleavage kinetics were quantified using FRET-based assays and demonstrated efficient processing of the substrate by matriptase under physiologically relevant conditions [33]. Despite expression of matriptase in H292, no activation of the prodrug was observed in the present study, as the inhibitory profiles of the cleavable prodrug and the non-cleavable control were indistinguishable (Fig. 9E). The results are consistent with previous reports showing that although H292 cells express active matriptase, the levels in monolayers are not sufficient for prodrug activation [20]. Fluorescence microscopy confirmed effective masking of the prodrug in multiple cell lines, including H292, FaDu, A431, and HaCaT cells expressing various levels of EGFR (Fig. 10). Notably, efficient masking was observed even in EGFR-overexpressing A431 cells, suggesting that the masking interaction remains sufficiently stable even in the presence of high receptor density. To mimic a more physiologically relevant setting, we evaluated prodrug binding in 3D spheroids (Fig. 11C). After 24 h incubation, H292 spheroids showed higher prodrug binding than FaDu, consistent with the presence of active matriptase in H292 but not FaDu, as previously reported by Vasiljeva and colleagues [46]. In vivo biodistribution studies in xenografted mice further supported matriptase activation as prodrug uptake in H292 tumors reached 57% of the cetuximab control, while in FaDu tumors uptake was only 32% of the cetuximab control (Fig. 11D).

While promising, further optimization may improve activation efficiency. For instance, incorporating more efficient cleavage sequences such as the recently reported improved matriptase substrate [33] could enhance prodrug activation. Notably, cetuximab is not cross-reactive with murine EGFR, so no conclusions can be drawn regarding potential blocking of EGFR binding in non-tumor tissue using this model. As with other conditionally activated biologics, systemic stability of the masked construct represents an important parameter for future translational development. In previous work, we performed an initial assessment of substrate specificity by evaluating cleavage of the here used matriptase substrates by other proteases, including uPA, legumain, and MMP-2, where no detectable processing was observed, suggesting limited susceptibility to off-target proteolysis. While the present study focused on demonstrating the feasibility of affibody-based masking and tumor-associated activation, more detailed analyses distinguishing intact and proteolytically cleaved antibody species in systemic circulation may provide additional insight into the stability of the prodrug format and will be of interest in future studies. Tumor microenvironments often exhibit heterogeneous protease expression profiles. Although matriptase represents a relevant tumor-associated protease in several epithelial cancers, activation efficiency may vary between tumor types. In the present study, we intentionally employed a single, well-characterized cleavage substrate to enable a controlled evaluation of the affibody-based masking concept while minimizing additional variables that could complicate mechanistic interpretation. While the use of a single substrate may provide improved specificity and reduce the risk of off-target activation, it may also limit activation efficiency in tumors with low matriptase expression. Future designs could therefore explore multi-protease-responsive linkers targeting additional tumor-associated proteases such as MMPs or cathepsins, although such strategies will require careful balancing of activation efficiency and substrate specificity.

In summary, we successfully established an *E. coli* display system for both the discovery and high-throughput characterization of specific antibody masking domains. Among the identified candidates, Zcet-1 emerged as the most promising, demonstrating effective masking when incorporated into a cetuximab prodrug, reducing the antibody’s functional activity by more than 400-fold in vitro.

The biodistribution results confirm the masking capacity of the newly discovered masking domain in vivo and indicated matriptase-mediated activation of the prodrug. The in vitro and in vivo data support the potential of affibody-based masking domains to fine-tune the activity of therapeutic antibodies, offering a flexible and modular approach to reduce systemic toxicity while maintaining efficacy. This work opens new avenues for the development of next-generation prodrugs for precision cancer therapy.

## Supplementary Information

Below is the link to the electronic supplementary material.

Supplementary Material 1

Supplementary Material 2

## Data Availability

No datasets were generated or analysed during the current study.
