# Supplementary material for: Engineering a conditionally active cetuximab prodrug via affibody-based paratope masking
Source: J Biol Eng. 2026 Jun 8;20:99. doi: 10.1186/s13036-026-00705-1 (PMC13248396; doi:10.1186/s13036-026-00705-1)
Supplement: Supplementary file 1 — Supplementary Material 1 [file 13036_2026_705_MOESM1_ESM.pdf]

SUPPLEMENTARY INFORMATION

Figure S1. Evaluation of single candidates by flow cytometry. A. *E. coli* single candidates isolated from FACS 3 and off rate libraries. Binding to cetuximab was evaluated by flow cytometry. **B.** The masking capacity of the candidates analyzed in (A) was evaluated by measuring the reduction in binding when the affibodies were incubated with the cetuximab-EGFR complex.

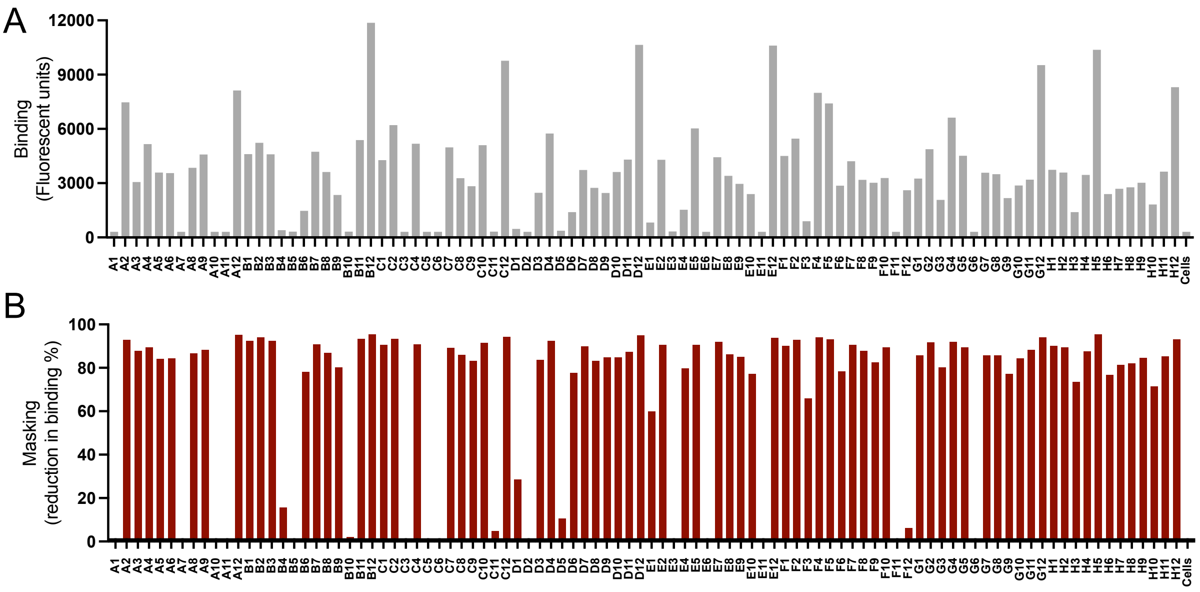

Figure S2. plDDT of complex predictions by AlphaFold3

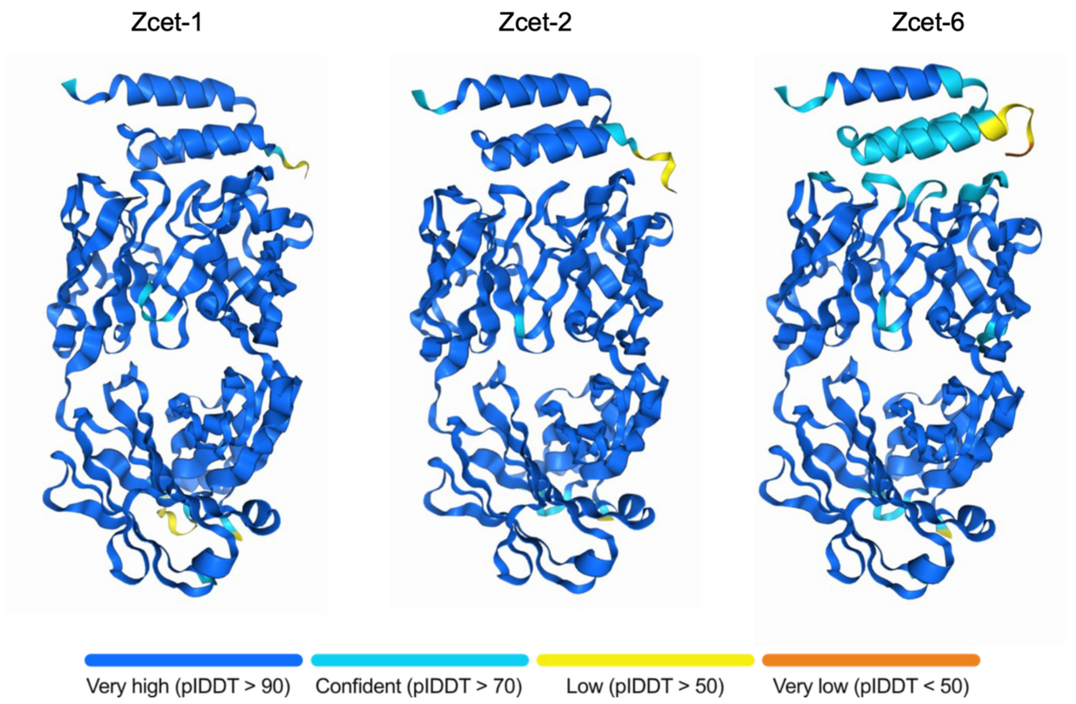

Figure S3. Size exclusion chromatography analysis of the purified prodrugs.

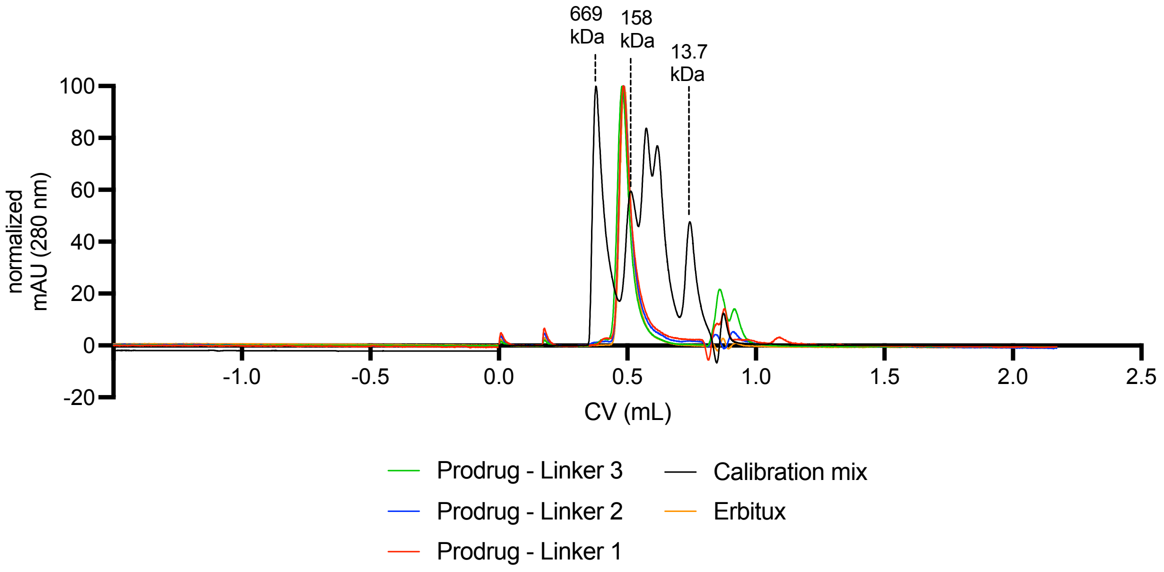

Figure S4. nanoDSF analysis of antibody constructs.
Unfolding transitions are shown as the first derivative of the intrinsic fluorescence ratio (F350/F330) with respect to temperature (dF/dT). Representative curves for the indicated constructs are shown, and the melting temperature (T_m) is indicated.

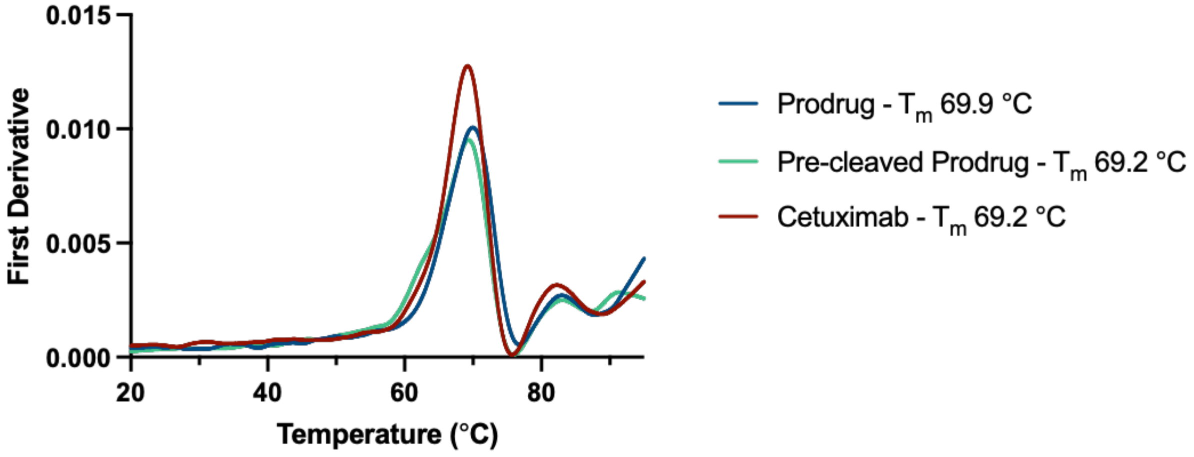

Supplementary Table 1. Radiolabeling results of cetuximab and cetuximab prodrug with indium-111. The data are presented as average ± standard deviation (SD) (n=3).

Compound	Radiochemical yield, %	Radiochemical purity, %	Maximal specific activity (MBq/µg)
**[^{111}In]In-cetuximab**	82 ± 18	99 ± 1	0.910
**[^{111}In]In-cetuximab prodrug**	65 ± 37	97 ± 1	0.230

Supplementary Table 2. Biodistribution of [^{111}In]In-cetuximab and [^{111}In]In-cetuximab prodrug in Balb/c nu/nu mice bearing H292 or FaDu xenografts 72 h p.i. The uptake of activity is expressed as percentage of injected dose per gram of tissue (%ID/g), except for skin, GI tract and carcass, where %ID is presented per whole sample. Results are presented as average from four mice ± SD.

Uptake, %ID/g				
	**H292**		**FaDu**	
	**[^{111}In]In-Cetuximab**	**[^{111}In]In-Cetuximab Prodrug**	**[^{111}In]In-Cetuximab**	**[^{111}In]In-Cetuximab Prodrug**
Blood	0.4 ± 0.1	4.3 ± 0.5	1.2 ± 0.6	1.6 ± 0.7
Salivary glands	1.1 ± 0.4	2.8 ± 1.2	1.5 ± 0.7	1.5 ± 0.7
Lungs	0.6± 0.4	2.0 ± 0.3	0.8 ± 0.4	1.0 ± 0.2
Liver	25 ± 9	12 ± 1	21 ± 10	26 ± 5
Spleen	4 ± 1	5 ± 2	4 ± 2	2 ± 1
Pancreas	0.16 ± 0.03	0.7 ± 0.3	0.3 ± 0.2	0.3 ± 0.2
Small intestine	0.3 ± 0.1	0.7 ± 0.1	0.5 ± 0.2	0.5 ± 0.4
Kidneys	1.8 ± 1.3	3.6 ± 0.3	1.5 ± 0.2	2.2 ± 0.7
Tumor	55 ± 7	31 ± 4	32 ± 12	10 ± 3
Muscle	0.2 ± 0.1	0.7 ± 0.2	0.2 ± 0.1	0.3 ± 0.1
Bone	0.4 ± 0.1	1.0± 0.2	0.6 ± 0.3	0.5 ± 0.1
Skin*	1.8 ± 0.6	3.7 ± 1.1	2.1 ± 0.8	4.5 ± 2.8
GI tract*	0.9 ± 0.1	1.9 ± 0.8	0.9 ± 0.2	0.9 ± 0.2
Carcass*	6 ± 1	21 ± 5	9 ± 4	13 ± 4

* %ID per whole sample.

Supplementary Table 3. Tumor-to-organ ratios of [111In]In-cetuximab and [111In]In-cetuximab prodrug in Balb/c nu/nu mice bearing H292 or FaDu xenografts 72 h p.i. Tumor-to-organ ratios were calculated only for tissues where uptake was expressed as %ID/g. Results are presented as average from four mice ± SD.

Tumor-to-organ ratio				
	**H292**		**FaDu**	
	**[^{111}In]In-Cetuximab**	**[^{111}In]In-Cetuximab Prodrug**	**[^{111}In]In-Cetuximab**	**[^{111}In]In-Cetuximab Prodrug**
Blood	159 ± 33	7 ± 1	37 ± 24	7 ± 2
Salivary glands	55 ± 22	12 ± 4	22 ± 8	8 ± 5
Lungs	115 ± 50	16 ± 3	44 ± 17	10 ± 2
Liver	2.4 ± 0.8	2.6 ± 0.2	2.4 ± 2.7*	0.4 ± 0.2
Spleen	16 ± 6	7 ± 3	10 ± 4	5 ± 1
Pancreas	360 ± 63	45 ± 15	114 ± 51	36 ± 12
Small intestine	221 ± 82	43 ± 4	78 ± 32	24 ± 8
Kidneys	40 ± 18	9 ± 2	21 ± 5	5 ± 1
Muscle	383 ± 103	52 ± 20	154 ± 62	36 ± 4
Bone	144 ± 22	33 ± 10	59 ± 16	23 ± 7

*Tumor-to-liver ratio showed high variability (mean 2.4 ± 2.7; n = 4), with individual values being 6,5; 1,1; 0,8; 1,3.
