# Supplementary material for: Engineering a conditionally active cetuximab prodrug via affibody-based paratope masking
Source: J Biol Eng. 2026 Jun 8;20:99. doi: 10.1186/s13036-026-00705-1 (PMC13248396; doi:10.1186/s13036-026-00705-1)
Supplement: Supplementary file 2 — Supplementary Material 2 [file 13036_2026_705_MOESM2_ESM.docx]

**SUPPLEMENTARY INFORMATION**

**Figure S1. Evaluation of single candidates by flow cytometry.** **A.** *E. coli* single candidates isolated from FACS 3 and off rate libraries. Binding to cetuximab was evaluated by flow cytometry. **B.** The masking capacity of the candidates analyzed in (A) was evaluated by measuring the reduction in binding when the affibodies were incubated with the cetuximab-EGFR complex.

**

**Figure S2.** **plDDT of complex predictions by AlphaFold3**


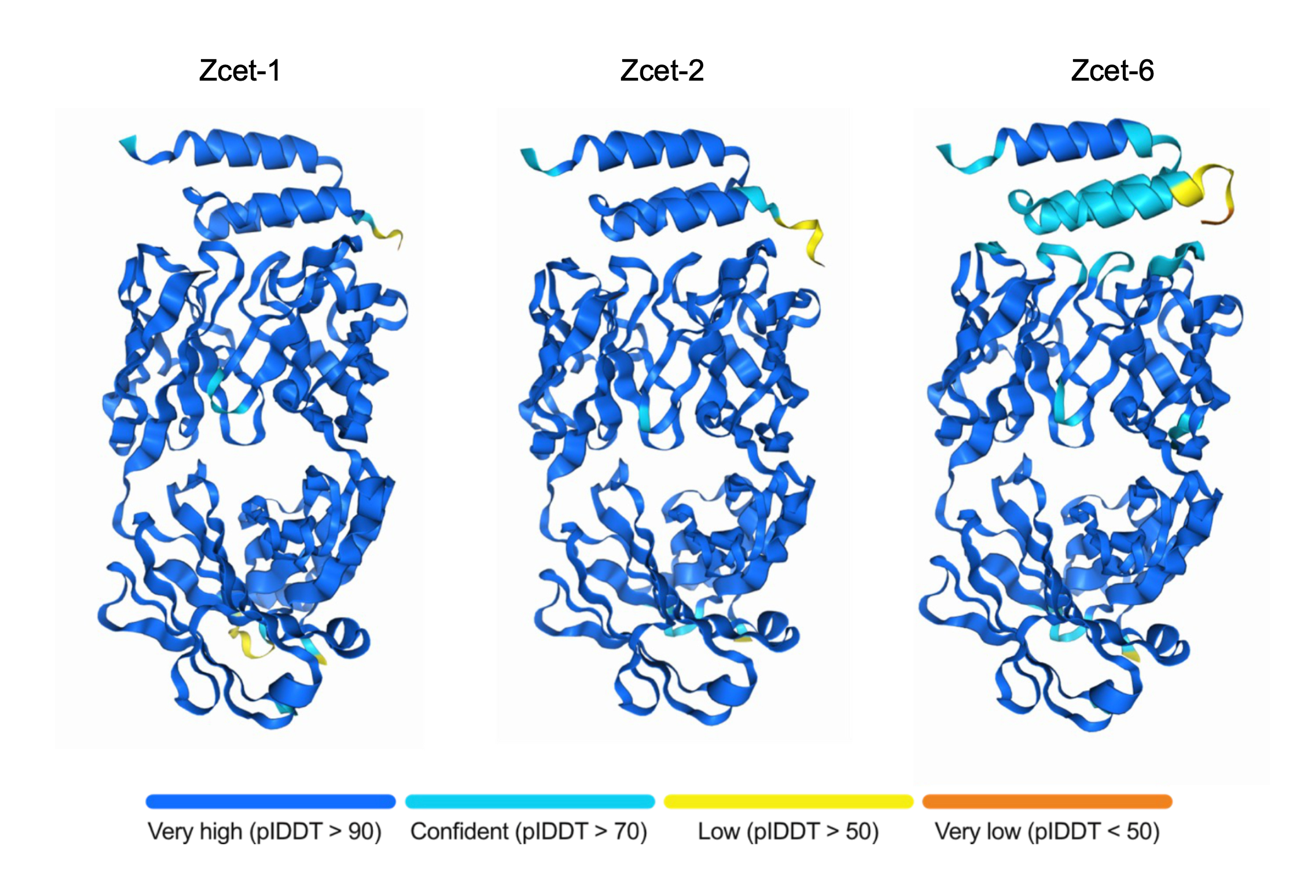


**Figure S3. Size exclusion chromatography analysis of the purified prodrugs.**

**Supplementary Table 1. Radiolabeling results of cetuximab and cetuximab prodrug with indium-111. The data are presented as average ± standard deviation (SD) (n=3).**

| Compound | Radiochemical yield, % | Radiochemical purity, % | Maximal specific activity (MBq/µg) |
| --- | --- | --- | --- |
| [^111^In]In-cetuximab | 82 ± 18 | 99 ± 1 | 0.910 |
| [^111^In]In-cetuximab prodrug | 65 ± 37 | 97 ± 1 | 0.230 |

**Supplementary Table 2. Biodistribution of [^111^In]In-cetuximab and [^111^In]In-cetuximab prodrug in Balb/c nu/nu mice bearing H292 or FaDu xenografts 72 h p.i. The uptake of activity is expressed as percentage of injected dose per gram of tissue (%ID/g), except for skin, GI tract and carcass, where %ID is presented per whole sample. Results are presented as average from four mice ± SD.**

| Uptake, %ID/g | | | | |
| --- | --- | --- | --- | --- |
|  | **H292** | | **FaDu** | |
|  | **[^111^In]In-Cetuximab** | **[^111^In]In-Cetuximab Prodrug** | **[^111^In]In-Cetuximab** | **[^111^In]In-Cetuximab Prodrug** |
| Blood | 0.4 ± 0.1 | 4.3 ± 0.5 | 1.2 ± 0.6 | 1.6 ± 0.7 |
| Salivary glands | 1.1 ± 0.4 | 2.8 ± 1.2 | 1.5 ± 0.7 | 1.5 ± 0.7 |
| Lungs | 0.6± 0.4 | 2.0 ± 0.3 | 0.8 ± 0.4 | 1.0 ± 0.2 |
| Liver | 25 ± 9 | 12 ± 1 | 21 ± 10 | 26 ± 5 |
| Spleen | 4 ± 1 | 5 ± 2 | 4 ± 2 | 2 ± 1 |
| Pancreas | 0.16 ± 0.03 | 0.7 ± 0.3 | 0.3 ± 0.2 | 0.3 ± 0.2 |
| Small intestine | 0.3 ± 0.1 | 0.7 ± 0.1 | 0.5 ± 0.2 | 0.5 ± 0.4 |
| Kidneys | 1.8 ± 1.3 | 3.6 ± 0.3 | 1.5 ± 0.2 | 2.2 ± 0.7 |
| Tumor | 55 ± 7 | 31 ± 4 | 32 ± 12 | 10 ± 3 |
| Muscle | 0.2 ± 0.1 | 0.7 ± 0.2 | 0.2 ± 0.1 | 0.3 ± 0.1 |
| Bone | 0.4 ± 0.1 | 1.0± 0.2 | 0.6 ± 0.3 | 0.5 ± 0.1 |
| Skin* | 1.8 ± 0.6 | 3.7 ± 1.1 | 2.1 ± 0.8 | 4.5 ± 2.8 |
| GI tract* | 0.9 ± 0.1 | 1.9 ± 0.8 | 0.9 ± 0.2 | 0.9 ± 0.2 |
| Carcass* | 6 ± 1 | 21 ± 5 | 9 ± 4 | 13 ± 4 |

* %ID per whole sample.
